# The Trends of Single-Cell Analysis: A Global Study

**DOI:** 10.1155/2020/7425397

**Published:** 2020-11-27

**Authors:** Hua Tian, Haifeng Liu, Yuanyuan Zhu, Dan Xing, Bin Wang

**Affiliations:** ^1^Department of Orthopedic, Xinyi People's Hospital, Xinyi, China; ^2^Department of Orthopedic, Second Hospital of Shanxi Medical University, Taiyuan, China; ^3^Pharmaceutical Department, Second Hospital of Shanxi Medical University, Taiyuan, China; ^4^Arthritis Clinic & Research Center, Peking University People's Hospital, Beijing, China; ^5^Department of Sports Medicine and Adult Reconstruction Surgery, Nanjing Drum Tower Hospital, The Affiliated Hospital of Nanjing University Medical School, Nanjing, China

## Abstract

**Objective:**

The field of single-cell analysis has rapidly grown worldwide, and a bibliometric analysis and visualization of data and publications pertaining to such single-cell research has the potential to offer insights into the development of this field over the past two decades while also highlighting future avenues of research.

**Methods:**

Single-cell analysis-related studies published from 2000-2019 were identified through searches of the Web of Science, Scopus, and PubMed databases, and corresponding bibliometric data were systematically compiled. Extracted data from each study included author names, country of origin, and affiliations. GraphPad Prism was used to analyze these data, while VOSviewer was used to perform global analyses of bibliographic coupling, coauthorship, cocitation, and co-occurrence.

**Results:**

In total, 4,071 relevant studies were included in this analysis. The number of publications increased substantially with time, suggesting that single-cell analyses are becoming increasingly more prevalent in recent years. Studies from the USA had the greatest impact in this field, with higher *H*-index values and numbers of citations relative to other countries, whereas Israel exhibited the highest average number of citations per publication. Bibliographic coupling, coauthorship, cocitation, and co-occurrence analyses revealed that Analytical Chemistry was associated with the highest number of publications in this field, and the University of Stanford contributed the most to this field. The most cited study included in this analysis was published by Macosko et al. in 2015 in *Cell.* Co-occurrence analyses revealed that the most common single-cell research topics included “mechanistic studies,” “*in vitro* studies,” “*in vivo* studies,” and “fabrication studies.”

**Conclusions:**

Single-cell analyses are a rapidly growing area of scientific interest, and higher volumes of publications in this field are expected in the coming years, particularly for studies conducting fabrication and in vivo single-cell analyses.

## 1. Introduction

Single-cell-based biological research is a complex but dynamic field [1, 2], enabling researchers to understand cellular phenotypes at single-cell resolution. Major advances in the fields of molecular biology, medicine, nanotechnology, and microfluidics have facilitated the proliferation of single-cell sequencing technologies. Historically, research in related fields was largely focused on specific applications such as DNA sequencing, transcriptomic profiling, or studies of chromatin accessibility. However, the spatial organization of cells within tissues is known to reflect functional differences in cell fate and lineage [3], and single-cell analysis thus represents an attractive approach to understanding such cellular heterogeneity and to understand its association with physiological homeostasis or disease [4]. These single-cell approaches also enable researchers to effectively analyze very rare cells including stem cells, circulating tumor cells (CTCs), and residual cells associated with a particular disease or therapeutic regimen [5]. Single-cell analyses allow for the integration of data pertaining to cellular genotype and transcriptional activity, thus offering more detailed information than that provided by traditional analytical techniques [6].

Owing to the power of this approach, single-cell research represents a rapidly expanding field of biological research [7] that has been leveraged for biochemical, diagnostic, and medicinal research. However, routine diagnostic use of this technology remains challenging at present [8].

Given the rapid growth of this research field, it is vital to explore how it has changed over time in order to predict future research trends. As such, we conducted a bibliometric analysis of single-cell research studies published over the past 20 years in an effort to quantitatively and qualitatively track the development of this field at a global level. Such bibliometric analyses focus on the contributions of individual research groups, institutes, journals, and countries to a given field of interest [9], and similar approaches have been used to help formulate clinical guidelines and policies aimed at improving the transparency of published research in recent years [10].

To the best of our knowledge, no prior bibliometric analyses pertaining to the field of single-cell analytical research have been conducted. This field is rapidly advancing and is associated with many new biological insights into microbial ecosystem diversity, the genomics of human single-cell malformation [11], animal cell deformities [12], plant cell diversity [13], and bacterial sporulation [14]. The goal and hypothesis of the present study were thus to analyze the current status of human single-cell research in time and to highlight relevant trends in order to proudly understand the global progress and future directions of this field.

## 2. Materials and Methods

### 2.1. Data Source

The present bibliometric analysis was conducted using the Web of Science (WoS) database, which compiles comprehensive citation and publication data across a range of scientific disciplines. In addition, the PubMed and Scopus databases were used to supplement these results.

### 2.2. Search Strategy

Two investigators (HT and BW) searched WoS in February 2020 in order to identify relevant studies published between 1 January 2000 and 30 December 2019 using the following search strategy: theme=“single-cell analysis” OR “single-cell analysis” OR “analyses, single-cell” OR “analysis, single-cell” OR “single-cell analyses” AND publishing year=(2000–2019) AND Document types=(review or article) AND Language=(English). Publication data pertaining to individual countries or regions was extracted by refining the corresponding search terms in WoS, Pubmed, and Scopus, with the following search terms (“Single-cell analysis” [Mesh Terms] OR “single-cell analysis” [All Fields] OR “analyses, single-cell” [All Fields] OR “analysis, single-cell” [All Fields] OR “single-cell analyses” [All Fields]) AND (“2001/01/01” [PDAT]: “2019/12/31” [PDAT]).

### 2.3. Data Collection

Complete WoS records pertaining to each study identified via the initial search strategy were downloaded, and two investigators (HT and BW) independently extracted data for each of these publications including author names, country of origin, affiliations, article title, year of publication, journal name, grants, keywords, and abstract. Any discrepancies were resolved through discussion or consultation with external experts. GraphPad Prism 8.0 was then used by both authors to independently analyze extracted data. Studies eligible for inclusion in this study were those pertaining to single-cell analysis including mechanistic studies, molecular biology analyses, medicine-related research, and microfluidics or nanotechnology articles. Studies were excluded from this analysis if they were not relevant, or if a corresponding full-text article was unavailable.

### 2.4. Bibliometric Analysis

Publication quality by country and author was assessed based upon metrics including total citations, average citations, and publication *H*-index values which were visualized using the WoS data visualization tool. *H*-index values serve as a measure of scientific impact for authors in different countries, whereas journal impact factor (JIF) values are used for individual publications. These values are computed based upon the number of published papers for a given author and the number of citations for each paper.

### 2.5. Visualized Analysis

VOSviewer (University of Leiden, Netherlands) was utilized to construct a bibliometric network visualization based upon coauthorship, bibliographic coupling, and cocitation analyses using extracted information including author names, affiliations, journals, and countries of origin. Co-occurrence networks were constructed to visualize important scientific terms represented within these publications.

## 3. Results

### 3.1. Global Publication Trends

#### 3.1.1. Global Publications by Country

**Figure 1:**
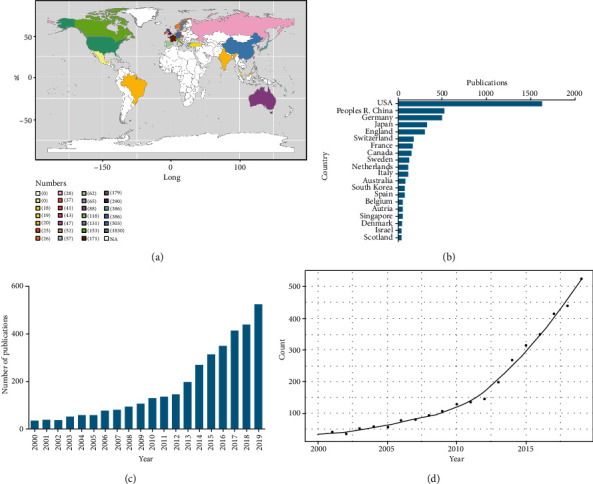
Global trends and countries contributing to single-cell analysis research. (a) World map showing the distribution of publications on single-cell analysis research. (b) The sum of related articles from the top 20 countries. (c) The single-year publication numbers in the past 20 years. (d) Model fitting curves of growth trends in worldwide publications and prediction of future publication numbers.

In total, 4,071 articles that met our search criteria were retrieved from WoS. Global contributions to the field of single-cell analysis research were then arranged in a color-coded map (Figure 1(a)), revealing that the United States (USA) contributed the greatest number of articles to this field (1,630; 40.04%), followed by the People's Republic of China (556; 13.66%), Germany (503; 12.36%), Japan (386; 9.48%), and England (290; 7.12%) (Figure 1(b)). In total, 3,251 and 3,567 articles were found through searches of PubMed and Scopus, respectively. Aggregated WoS data were used for subsequent bibliographic coupling, coauthorship, and cocitation analyses in VOSviewer, as discussed below.

#### 3.1.2. Global Publications by Year

The year with the highest number of publications in this field was 2019 (525; 12.90%), and the overall trajectory from 2000 to 2019 exhibited exponential growth with respect to the global number of single-cell-related publications (Figure 1(c)).

#### 3.1.3. Prediction of Global Publication Trends

We next used a logistic regression model to predict future publication trends in this field (Figure 1(d)), indicating that while rates of publication in this field are currently in a stage of rapid growth, this growth rate is likely to decline over the coming decade.

#### 3.1.4. Grant Support Trends

United States Department of Health Human Services supported the greatest number of publications in this analysis (800), followed by the National Institutes of Health (NIH, USA) (798), the National Natural Science Foundation of China (504), the Ministry of Education Culture Sports Science and Technology (Japan) (142), and German Research Foundation DFG (130).

#### 3.1.5. Publication Classifications

Of the identified publications, 946 were classified as chemistry studies, followed by biochemistry molecular biology (906), science technology other topics (640), cell biology (412), and biotechnology/applied microbiology (276).

### 3.2. Quality of Studies from Different Countries and Authors

#### 3.2.1. Total Citation Frequency

**Figure 2:**
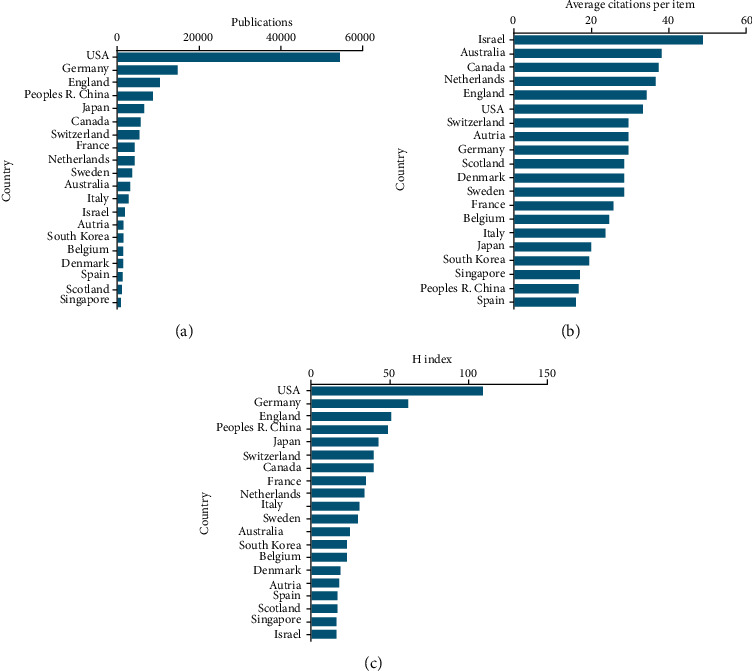
Citation frequency and *H*-index of different countries publishing in single-cell analysis research. (a) Total citations of research articles from different countries. (b) Average citations per article from different countries. (c) *H*-index of publications from different countries.

American publications exhibited the greatest number of citations in this study (373,576), followed by China (221,460), Germany (125,405), England (86,246), and Japan (77,142) (Figure 2(a)).

#### 3.2.2. Average Citation Frequency

Studies from Israel exhibited the highest average number of citations per publication (48.92), followed by Australia (38.17), Canada (37.56), Netherlands (36.67), and England (34.37) (Figure 2(b)).

#### 3.2.3. *H*-Index for Countries

Publications from the USA exhibited the highest average *H*-index (109), followed by Germany (62), England (51), China (49), and Japan (43) (Figure 2(c)).

#### 3.2.4. *H*-Index for Authors

Publications from Christopher J Love exhibited the highest *H*-index values (20), followed by Jin Wenrui (12), Kitamori Takehiko (10), Chen Jian (9), and Wang Joseph (9).

### 3.3. Bibliographic Coupling Analysis

A bibliographic coupling analysis was used to analyze links between publications and the references cited in those publications. When two publications referenced the same article, they were considered to be “coupled”, suggesting that these articles shared a common theme. The strength of the link between two publications was measured based upon the number of common references cited by both publications. This analytical approach was used to gauge link strength among journals, countries, and institutions that had published single-cell sequencing analyses.

#### 3.3.1. Journals

**Figure 3:**
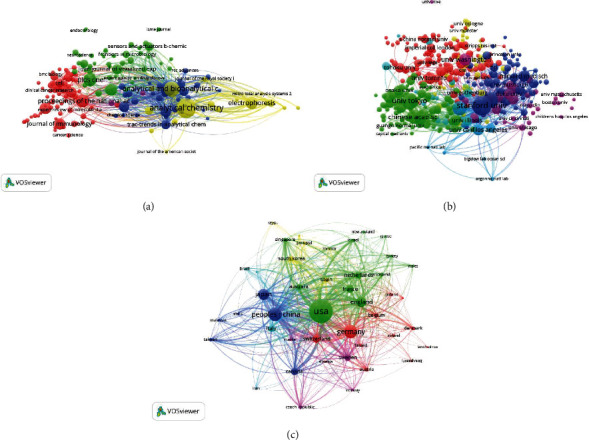
Bibliographic coupling analysis of global research on single-cell analysis. Mapping of (a) 175 identified journals, (b) 384 institutions, and (c) 44 countries on the research area. The line between two points in the map indicates that two journals/institutions/countries had established a similarity relationship. The thicker the line, the closer the link between the two entities.

A total of 175 journals were identified (Figure 3(a)). The top 5 journals with the greatest link strength values were *Analytical Chemistry* (2018 impact factor = 6.35; total link strength = 31,827 times), *Lab on a Chip* (6.914; 30,088), *Analyst* (4.019; 19,733), *Analytical and Bioanalytical Chemistry* (3.286; 17,158), and *Electrophoresis* (2.754; 13,080).

#### 3.3.2. Institutions

**Figure 4:**
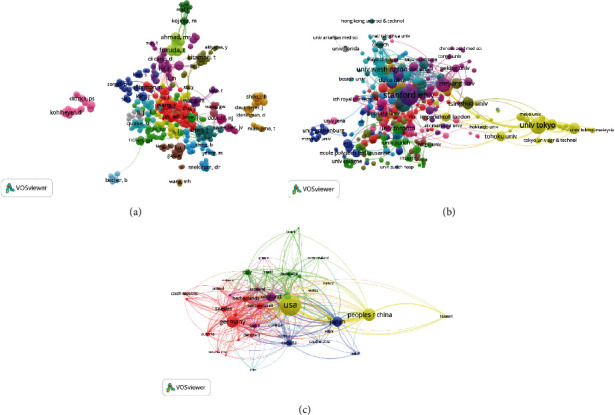
Coauthorship analysis of global research on single-cell analysis. Mapping of (a) 392 authors, (b) 384 institutions, and (c) 44 countries on the research area. The size of the points represents the coauthorship frequency. The line between two points indicates that two authors/institutions/countries had established collaboration. The thicker the line, the closer the collaboration between the two entities.

In total, 384 institutions were identified through this analysis (Figure 4(b)). The institutions with the highest linkage strengths were Stanford University (total link strength = 66,715), Harvard University (64,155), MIT (50,922), Chinese Acad SCI (42,959), and Tsinghua University (38,234).

#### 3.3.3. Countries

In total, 44 countries were identified through this analysis (Figure 3(c)). The top 5 nations with the highest linkage strengths were the USA (total link strength = 373,576), China (211,460), Germany (125,405), England (86,246), and Japan (77,142).

### 3.4. Coauthorship Analysis

A coauthorship analysis was conducted to measure interconnectivity between researchers, with the strength of these connections being measured based upon the number of publications coauthored by a given pair of researchers. This approach was used to not only assess the strength of links between authors but also between countries and institutions.

#### 3.4.1. Authors

In total 77 authors that had published a minimum of 10 articles were identified through this analysis (Figure 4(a)). The top 5 authors by total link strength were Fukuda T (total link strength = 83), Nakajima M (83), Homma M (69), Kojima S (66), and Chen Dy (53).

#### 3.4.2. Institutions

We analyzed organizations that had published more than 10 articles (Figure 4(b)). The top 5 institutions that had the highest linkage strengths were Harvard University (total link strength = 175), Harvard Medical School (123), Stanford University (122), MIT (117), and Dana Farber Cancer Institute (94).

#### 3.4.3. Countries

A total of 57 countries had generated a minimum of 10 single-cell sequencing publications over the past decade (Figure 4(c)). The nations with the highest total linkage strengths were the USA (total link strength = 685), Germany (336), England (281), China (246), and France (165).

### 3.5. Cocitation Analysis

A cocitation analysis was performed to assess the connection between publications that were cited in other publications, with total link strength being calculated based upon the total number of publications in which specific publications were cited together. This same approach was also used to assess cocitation linkage strength between authors and journals.

#### 3.5.1. Authors

**Figure 5:**
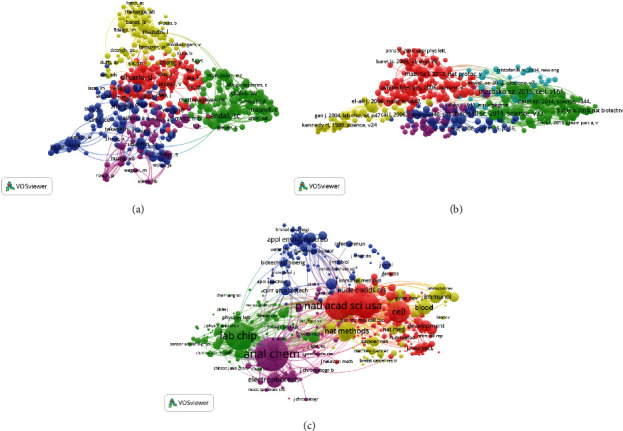
Cocitation analysis of global research on single-cell analysis. Points with the same color belong to the same research topic. (a) Mapping of cocited authors in the field. Points with different colors represent the 2575 cited authors. (b) Mapping of cocited references in the field. Points with different colors represent the 1385 cited references. (c) Mapping of cocited journals in the field. Points with different colors represent the 1453 identified journals. The size of the points represents the citation frequency. A line between two points means that both were cited in one paper or journal. A shorter line indicates a closer link between two entities.

In total, we analyzed 901 authors that were cocited in over 20 publications (Figure 5(a)). The top 5 authors with the highest total linkage strength values were DiCarlo D (total link strength = 6,189), Bendall SC (5,455), Mazutis I (5,183), Tang FC (4,882), and Rubakhin SS (4,287).

#### 3.5.2. References

In total, 430 references were cited in 20 or more publications (Figure 5(a)). The top 5 references with the highest total linkage strength values were Macosko EZ, 2015, *Cell* (total link strength = 2,674 times), Klein AM, 2015, *Cell* (2,425), Bendall SC, 2011, *Science* (2,040), Tang FC, 2009, *Nature Methods* (1,953), and Brouzes E, 2009, *PNAS* (1,670).

#### 3.5.3. Journals

Journals were analyzed for references cited in all publications, and total cocitation linkage strengths were computed for 919 total journals (Figure 5(c)). The top 6 journals with the highest link strength values were *Analytical Chemistry* (total link strength = 724,293 times), *Lab on a Chip* (572,258), *Proceedings of the National Academy of Sciences of the United States of America* (501,086), *Science* (441,896), *Nature* (439,147), and *Cell* (283,338).

### 3.6. Co-Occurrence Analysis

**Figure 6:**
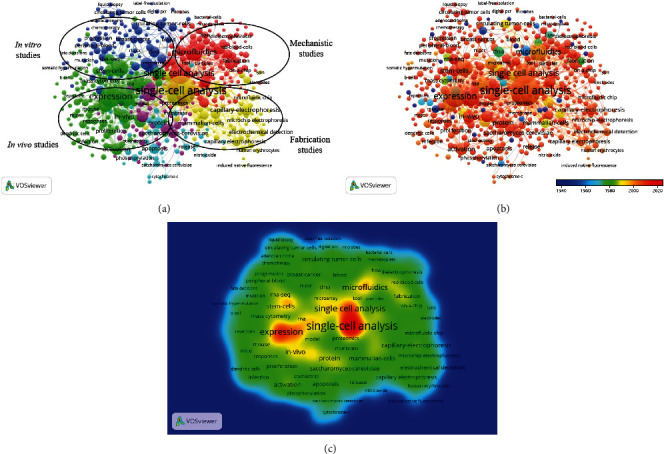
Co-occurrence analysis of global research on single-cell analysis. (a) Mapping of keywords in the research area. The size of the points represents the frequency of appearance, and the keywords are divided into four clusters: mechanistic studies (red), in vitro studies (blue), in vivo studies (green), and fabrication studies (yellow). (b) Distribution of keywords according to the chronological order of appearance. Keywords in blue appeared earlier than those in yellow and keywords in red appeared the latest. (c) Distribution of keywords according to the mean frequency of appearance. Keywords in red occurred with the highest frequency, followed by yellow, green, and cyan.

A co-occurrence analysis was additionally conducted in order to analyze the number of publications in which specific keywords occurred simultaneously as a means of gaining insight into future directions in the field of single-cell research. In this study, 1273 keywords were used over 5 times, and these keywords were broadly separated into four clusters: “*in vivo* studies,” “*in vitro*,” “mechanistic studies,” and “fabrication studies” (Figure 6(a)). These clusters encompassed the majority of published single-cell research to date.

Relevant keywords were coded using VOSviewer based upon the year of publication (Figure 6(b)), with keywords in earlier studies appearing in blue and keywords in more recent studies appearing in red. The majority of articles published before 2014 were “mechanistic studies” and “in vitro studies”, whereas “in vivo studies” and “fabrication studies” have become more common in recent years and are likely to remain major areas of research in the future (Figure 6(c)).

## 4. Discussion

### 4.1. The Impact of Single-Cell Analysis on Modern Biomedical Research

Diversity within a given unicellular species or population of heterogeneous cells is complex and cannot be fully captured by aggregate analyses of mixtures of cells. As such, single-cell research has enabled far more in-depth biomedical studies to be conducted. For example, researchers can now define the contributions of individual cells to intratumoral heterogeneity and therapeutic responses in the context of cancer [15]. However, these analyses can at times be limited by insufficient genomic coverage or inadequate numbers of loci [16]. Research efforts have thus focused on the amplification and analyses of large numbers of cells and on the development of standardized approaches to determining cancer clonal structures and guiding single-cell analyses [17]. The Human Tumor Atlas Network (HTAN), which is a part of the National Cancer Institute (NCI) Cancer Moonshot Initiative, seeks to chart tumor spatiotemporal tumor transitions with single-cell resolution [18].

### 4.2. Research Trends in Single-Cell Research

We employed several different visual and bibliometric analyses to assess the current status of the single-cell research, analyzing the relative contributions of specific authors, institutions, journals, and nations to this dynamic field. We additionally highlighted research topics that are likely to be a major research focus in the near future. Since the inception of this field in 1994, there have been exponentially increasing volumes of single-cell research that has been most pronounced over the past 3-4 years. This is attributable to the unparalleled advantages of single-cell analyses, which enable researchers to probe cell-specific heterogeneity that cannot be evaluated using more traditional techniques [8]. Over 3000 articles in this study were published since 2013. Significant bibliographic coupling was observed for publications of institutions in over 75 countries, underscoring the strength and breadth of knowledge in this field over the past 20 years. These trends suggest that single-cell research will be a central focus of future biological research, and our co-occurrence analyses additionally suggest that the number of fabrication and in vivo single-cell studies is likely to rise in the near future.

### 4.3. Quality of Global Publications by Institution, Country, and Journal

The USA is currently the world leader in the field of single-cell-based research, with studies from the USA being ranked first with respect to total numbers of citations, *H*-index values, coauthorship analyses, and bibliographic coupling analyses. These results suggest that these publications may have a major impact on the overall quality and direction of research in this field. Over the past decade, the quality and impact of studies conducted in China, Britain, Germany, Switzerland, and other countries have also risen substantially.

Over the past decade, the number of single-cell analysis papers in China has risen rapidly such that China now ranks second in the world with respect to the total number of papers in this field. However, these Chinese studies were only ranked 4^th^, 19^th^, and 4^th^ with respect to total citations, average citations, and *H*-index values, respectively.

Differences in the quantity, quality, and impact of these studies are at least partially attributable to cultural differences between the academic systems in China and Western nations. The Chinese system primarily emphasizes the number of publications rather than their underlying quality [19]. However, as research funding in China has grown over recent years, gradual improvements in publication quality have been observed, and these trends are likely to continue over the coming years and to align with global publication trends.

Institution-based bibliographic coupling and coauthorship analyses in this study reflected the relative contributions of particular research institutions to the overall field of single-cell research. Consistent with country-based trends, institutions from the USA and China were among the top contributors to this field. The top 5 institutions identified via both bibliographic coupling, and coauthorship analyses indicated that Stanford University and Harvard University in the USA were ranked higher than were the Chinese Academy of Science and the Tsinghua University in China. Overall, these findings suggest that the academic output of a given research institution is closely tied to the ranking of the country in which that institution is located, with the top 5 such institutions being those with the greatest global authority in this research field.

Coauthorship and cocitation analyses enabled us to identify one author who was in the top 5 rankings for both of these analyses. Highly ranked authors may improve the overall ranking of institutions with which they are affiliated in the field of single-cell analysis research.

Journal link strengths in our bibliographic coupling and cocitation analyses indicated the degree to which a given journal is relevant to the field of single-cell research, while also providing an indirect ranking of journals within this field. In addition, cocitation analyses revealed that fundamental cornerstone analyses in this field with the highest cocitation link strength values markedly bolstered the impact values of the journals in which these studies had been published. Together with the results of our bibliographic and cocitation analyses, we determined that *Analytical Chemistry* is the most prolific journal in this field, followed by *Lab on a Chip* and *PNAS.*

### 4.4. Future Outlook

Our co-occurrence network diagram highlighted likely future single-cell analysis research directions. Broadly speaking, these research directions included mechanistic research, in vitro research, in vivo research, and fabrication research, with a number of individual topics being associated with each of these fields (Figure 6(a)). Single-cell mechanistic and fabrication analyses have the potential to guide the development of novel therapies, point-of-care diagnostics, and complex microfluidic platforms, making it possible to prepare in vitro cellular microenvironments well suited to drug and toxicity screening [20]. With respect to in vivo analyses, efforts to utilize in vitro engineered cell lines in a complex live animal model system have historically been confounded by cell type heterogeneity. While identifying these cells via flow cytometry that has met with some success, it generally necessitates prior knowledge of appropriate cellular markers and characteristics. These limitations have led to an increasing research focus on the use of a range of single-cell analytical techniques when conducting in vivo studies. For example, Cusanovich et al. utilized single-cell combinatorial indexing-ATAC-seq to generate a single-cell atlas of in vivo/in vitro mammalian chromatin accessibility [21]. Since the publication of the first single-cell RNA-seq study in 2009 [22], many researchers have leveraged and expanded upon this technology to compare individual cellular transcriptomes as a means of better understanding and controlling for heterogeneity within and among cellular populations [23].

This co-occurrence analysis also identified a number of “remote” research themes that did not cluster with other research topics owing to a lack of strong co-occurrence linkages. These “remote” themes included chemotherapy, microfluidic chips, and circulating tumor cells. While these themes may not be representative of the field as a whole, they nonetheless seek to answer important research questions and warrant future study.

The organization of subject areas by publication data offered useful insights into past and future trends in the field of single-cell analyses (Figure 6(b)). Notably, many recent publications included keywords that led them to be grouped under the “fabrication” and “in vivo” clusters, indicating that future research is likely to focus on these topics. Some of these emergent research topics also overlapped with other “remote” keywords in order studies including microfluidic chips, peripheral blood analyses, and capillary electrophoresis, suggesting that single-cell research efforts in these fields may continue to expand rapidly in the near future.

### 4.5. Strengths and Limitations

Herein, we conducted a comprehensive survey of the literature to perform quantitative, qualitative, and visual analyses of publications pertaining to single-cell research in an effort to understand the current and future state of this research field. While this analytical approach is powerful, there are nonetheless several limitations to our analyses. For one, we only included studies published in English in this analysis, potentially introducing selection bias owing to the exclusion of studies published in other languages. Second, recent publications, even when published in high-profile journals, typically have fewer citations than do studies published several years ago. As such, recent publications are likely to have been underweighted in our analyses due to this time effect.

## 5. Conclusions

Our data indicate that the USA is a major contributor to the field of single-cell research. The number of publications in this field is expected to continue to grow rapidly in the coming years, as interests increasingly shift into multidisciplinary research areas. In particular, future studies leveraging single-cell analytical approaches for fabrication and in vivo research are predicted to be prevalent and to advance the field as a whole.

## Abbreviations

DNA: Deoxyribonucleic acid
RNA: Ribonucleic acid
CTC: Circulating tumor cells
WoS: Web of science
JIF: Journal impact factor.
